# A Step-by-Step Conservative Approach for CAD-CAM Laminate Veneers

**DOI:** 10.1155/2017/3801419

**Published:** 2017-08-13

**Authors:** Gerardo Durán Ojeda, Ismael Henríquez Gutiérrez, Álvaro Guzmán Marusic, Abelardo Báez Rosales, José Pablo Tisi Lanchares

**Affiliations:** ^1^Health Sciences Faculty, Universidad Arturo Prat, Iquique, Chile; ^2^Restorative Dentistry Department, Andrés Bello University, Viña del Mar, Chile

## Abstract

The use of CAD/CAM technology has allowed the fabrication of ceramic restorations efficiently and with predictable results. Lithium disilicate is a type of glass ceramic material that can be used for the elaboration of laminate veneers, being monolithic restorations which require characterization through a covering ceramic in order to achieve acceptable esthetic results. The next case report shows a predictable clinical protocol for the rehabilitation of the anterior teeth through the preparation of CAD/CAM veneers (e.max CAD, Ivoclar Vivadent, Liechtenstein) which have been characterized by a nanofluorapatite ceramic (e.max Ceram, Ivoclar Vivadent, Liechtenstein) through the layering technique.

## 1. Introduction

Glass ceramic materials have been widely used to restore enamel loss due to its mechanical and optical properties [1, 2]. This restorative material, used for the indirect method, can be processed by traditional laboratory procedures, which is a highly sensitive technique or CAD/CAM technology [3, 4].

The use of CAD/CAM nowadays is a useful tool that allows digital impression taking, digital design as part of the treatment planning, and the elaboration of monolithic restorations for ceramic materials, used most recently in the field of ceramic veneers [5–7].

For the elaboration of ceramic laminate veneers, lithium disilicate is a ceramic reinforced material which can be processed by CAD/CAM systems or through the press technique. In case of CAD/CAM, this material is presented as blocks of lithium metasilicate in a precrystallized state which contains cores of lithium metasilicate and disilicate, reducing its flexural strength to 130 ± 30 Mpa. This allows the milling process, in which the shape of the restoration is obtained. Then, the ceramic is recrystallized at 850°C for 20 to 25 minutes. During this procedure, the lithium metasilicate dissolves and the lithium disilicate crystalizes, obtaining the final translucency of the restoration. Finally, the quantity of crystals and the flexural strength increases to 70% in volume and to 360–400 Mpa, respectively [3, 8].

At this point, the final restoration may not have the ideal tooth optical properties. In this case, there are three methods for the characterization of the incisal third: (a) cut-back technique; (b) staining technique; and (c) layering technique.

The present case report shows a step-by-step procedure of CAD/CAM lithium disilicate veneers characterized with a final layering technique with nanofluorapatite ceramic.

## 2. Case Report

### 2.1. Case Presentation

**Figure 1:**
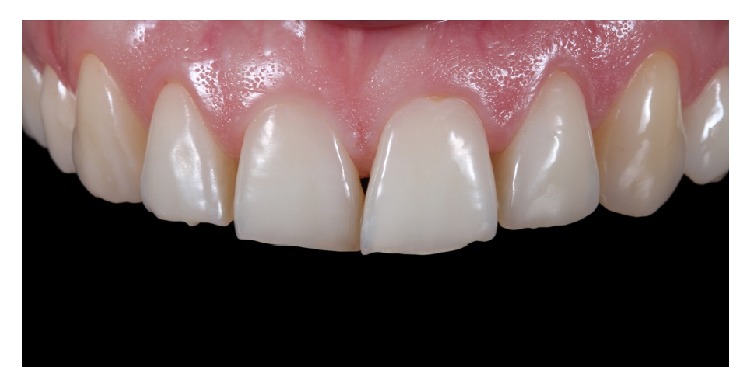
Intraoral image, showing disharmony in position and size, dental attrition, and erosion.

A 27-year-old female patient presented to the private practice of one of the authors with a main complain of dental erosion and attrition and size disharmony of the maxillary anterior dentition (Figure 1).

### 2.2. Treatment Planning

**Figure 2:**
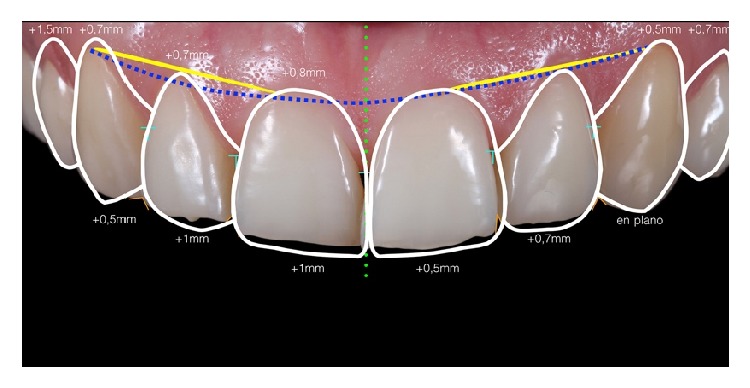
Digital smile design (DSD). Planning of the anterior teeth proportions for the wax-up.

**Figure 3:**
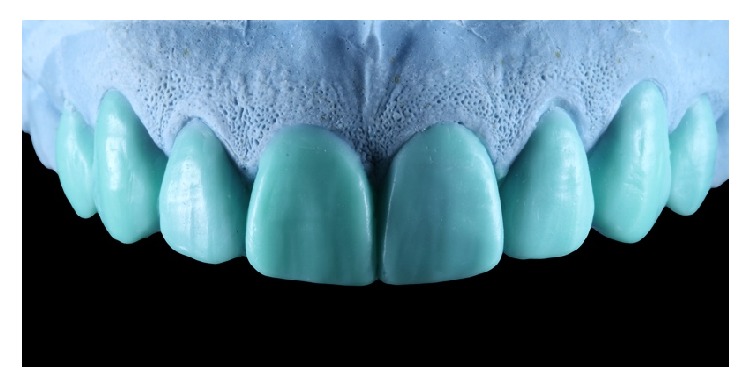
Wax-up, identical in form from the DSD.

**Figure 4:**
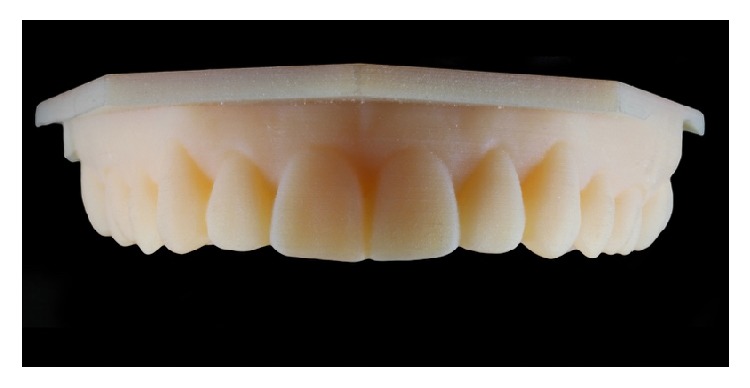
3D resin model obtained from the wax-up scanning.

**Figure 5:**
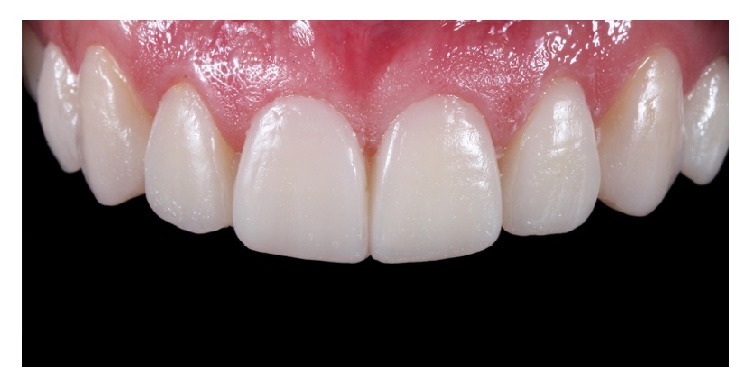
Mock-up with bisacrylic resin (Luxatemp Star A1, DMG). Notice the shape, which is the same as the DSD planning.

After a thorough clinical examination, a digital smile design was performed to decide the correct anterior tooth proportions (Figure 2) which includes tooth 14 to tooth 24. This design was transferred to a diagnostic wax-up in which the enamel loss could be appreciated by the amount of wax over the cast model (Figure 3). Once the wax-up was done, it was digitalized with a model scanner (dental wings 3 series) and printed into a 3D resin model (PASTCure Model 310) (Figure 4), in which a silicone matrix was fabricated for the mock-up technique. The mock-up was done with bisacrylic material (Luxatemp Star A1, DMG) and is shown in Figure 5.

### 2.3. Tooth Preparations

**Figure 6:**
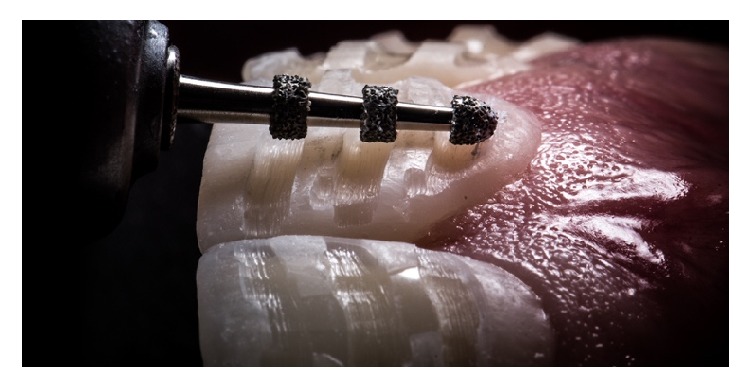
Orientation grooves for the vestibular reduction.

**Figure 7:**
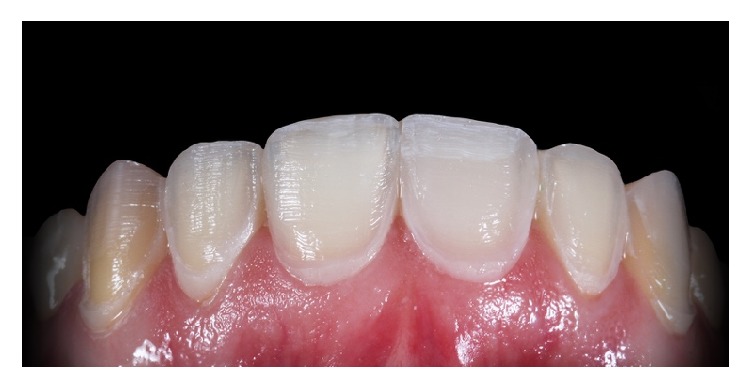
Vestibular reduction until the painted grooves are removed; this ensures a controlled reduction for the ceramic space.

**Figure 8:**
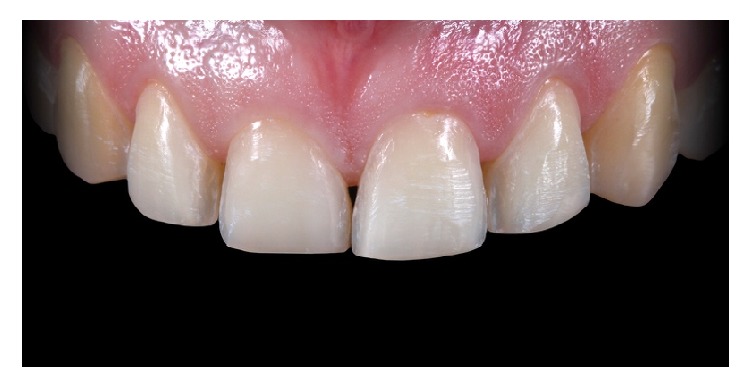
Finished tooth preparations.

Once the mock-up was approved by the patient, the preparations were performed. This was made using the mock-up as a guide for a controlled preparation [9]. A diamond bur kit for laminate veneers was used (Ceramic Laminate Veneers Kit. Ref. 9933K3 000, LOT. 797593, Komet). The first step includes the creation of orientation grooves (868B.314.018, Komet) for the vestibular reduction (Figure 6) through the bisacrylic material. Every groove was marked with a graphite pencil. Then, a tapered shape bur was used to complete vestibular reduction (6850.314.016, Komet) until every marked groove was removed (Figure 7). 1,5 mm grooves for the incisal reduction were done, followed by the proximal preparation, which was made with an 8850.315.016 tapered diamond bur, and finally, a diamond disc (952.180 + 310.204, Komet) was necessary for the elimination of the contact point between 11 and 21 tooth due to a misalignment problem. An image for the all tooth preparations after polishing can be shown in Figure 8.

### 2.4. Impression Procedure and Fabrication of Laminate Veneers

**Figure 9:**
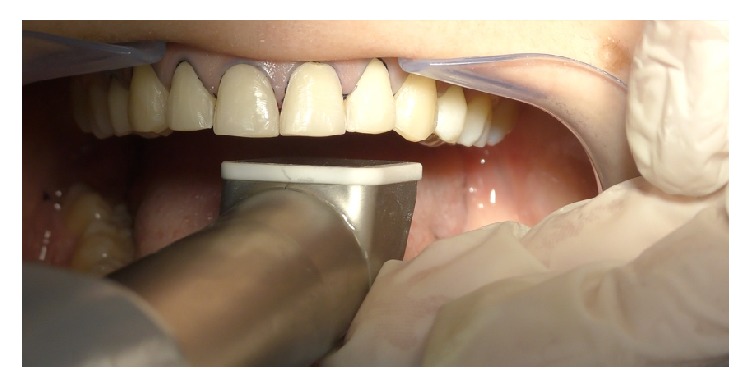
Intraoral scanning.

**Figure 10:**
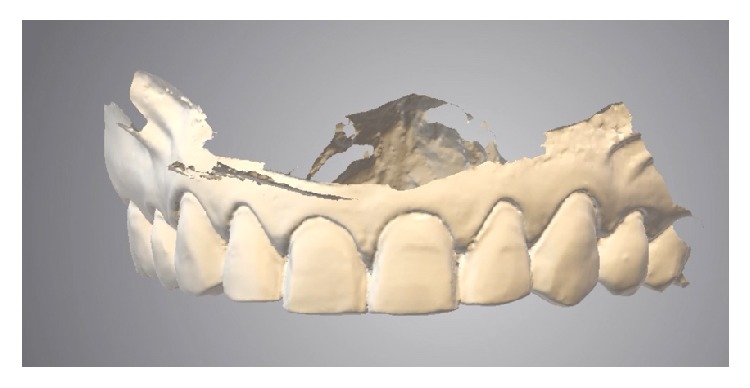
Digital model with the finished preparations for the ceramic digital design.

**Figure 11:**
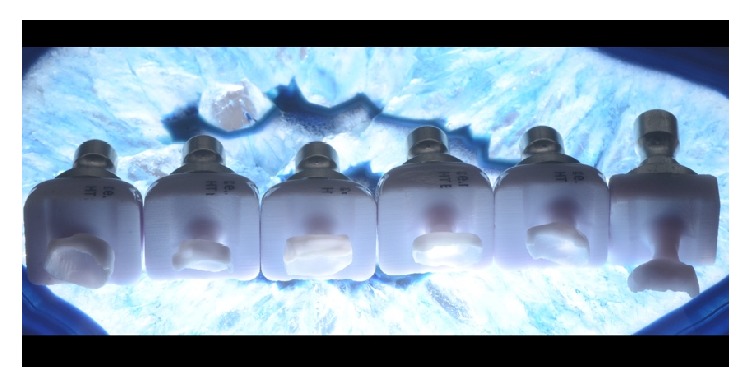
Milled e.max CAD blocks.

**Figure 12:**
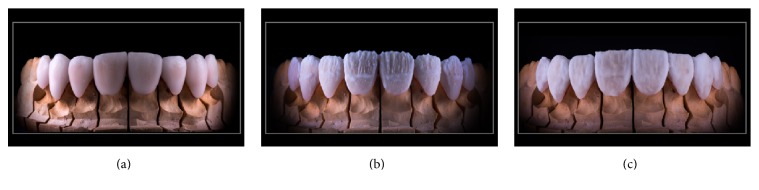
Ceramic layering technique. (a) The fitting of the veneers is being checked. (b) Power Dentin (IPS e.max Ceram) is being applied. (c) A final layer application of Power Enamel (IPS e.max Ceram).

**Figure 13:**
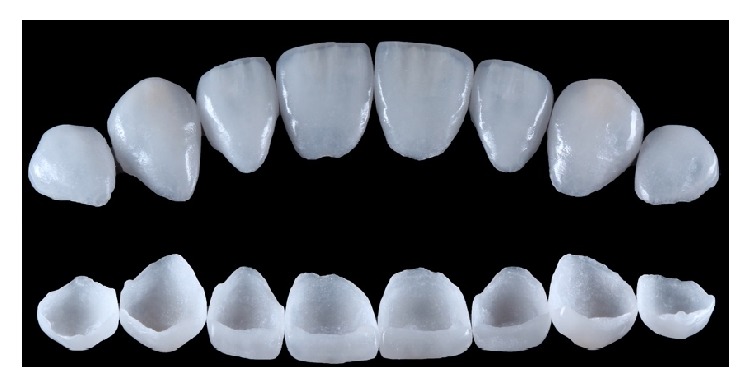
Finished ceramic veneers and vestibular and intaglio surface view.

A retraction cord was placed into every sulcus of the prepared tooth (000, Ultrapak, Ultradent Products Inc.). 3D digital impression was performed using an optical intraoral scanner (MHT IntraOralScanner, MHT Optic Research, Zurich, Switzerland) and then digitalized (Figures 9 and 10). The software used for the restorations design process was ExoCad® DentalCAD (White Peaks Dental Systems GmbH & Co. KG), which created eight veneer restorations from tooth 14 to tooth 24. The milling unit used in this case was Roland DWX-4W (DWX-4W, Roland Easy Shape Dental Solutions, Australia), which milled eight IPS e.max® CAD HT-A1 blocks (Ivoclar Vivadent, Liechtenstein, Germany). The immediate result of the milling process is shown in Figure 11. Then, every milled block was layered with a nanofluorapatite ceramic for the final incisal edge optics (IPS e.max Ceram, Ivoclar Vivadent, Liechtenstein, Germany) (Figure 12). The final result of the sintering and glaze procedure is shown in Figure 13.

### 2.5. Try-In Procedure

**Figure 14:**
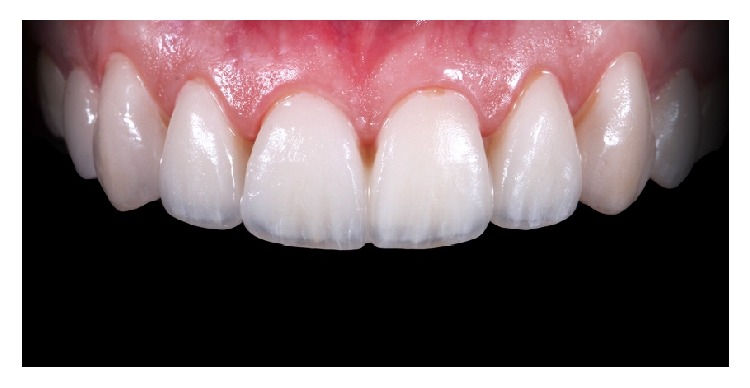
Try-In +2 value.

Each restoration was tested into their corresponding preparation for fitting. Try-in pastes were used to select the color of the resin cement, always in consideration with the patient needs and expectations. In this case, value +2 of Variolink Veneer resin cement system was used (Try-In +2, Variolink Veneer, Ivoclar Vivadent, Liechtenstein, Germany) (Figure 14).

### 2.6. Cementation Procedure

The treatment surfaces can be divided into two: (a) the treatment surface of the laminate veneers and (b) the treatment surface of the tooth preparations. This procedure was done under rubber dam isolation, which ensures the quality of bonding and allows clean and visible surfaces for cementation.

#### 2.6.1. Treatment Surface of the Laminate Veneers

**Figure 15:**
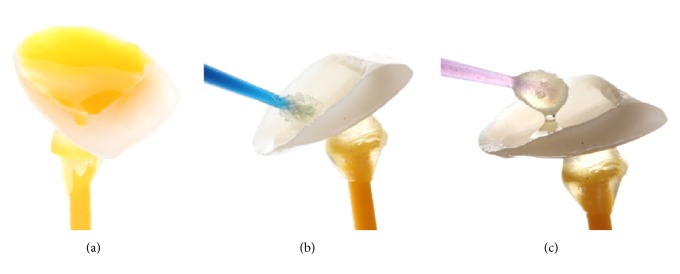
Ceramic treatment surface. (a) The conditioning of the veneer with 5% hydrofluoric acid for 20 seconds. (b) Silane application (Monobond Plus, Ivoclar Vivadent, Liechtenstein, Germany). (c) Application of bonding agent (Heliobond, Ivoclar Vivadent, Liechtenstein, Germany).

The treatment of the intaglio surface of the lithium disilicate veneers starts with the partial dissolution of the ceramic glass content with 5% hydrofluoric acid for 20 seconds (Power C Etching 5%, BM4, Florianópolis, Brazil), then the acid was rinsed off with an air water spray for 30 seconds [10], and silane coupling agent was applied in a thin layer (Monobond Plus, Ivoclar Vivadent, Liechtenstein, Germany) and heated at 100°C for 1 minute. A final thin layer of bis-GMA bonding agent was applied (Heliobond, Ivoclar Vivadent, Liechtenstein, Germany) without light curing and protected from the light (Vivapad, Ivoclar Vivadent, Liechtenstein, Germany) (Figure 15).

#### 2.6.2. Treatment Surface of the Prepared Tooth

**Figure 16:**
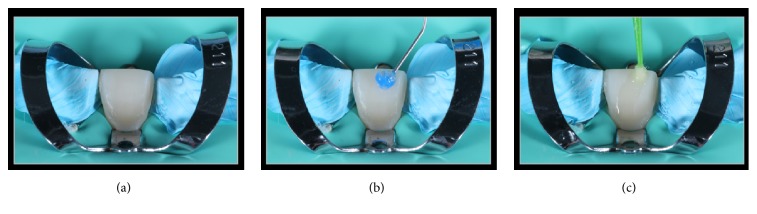
Treatment of the enamel surface for bonding. (a) Tooth 11 under rubber dam isolation. (b) 37% phosphoric acid etching. (c) Bonding application.

All surfaces of prepared tooth were in enamel. A phosphoric acid etching gel at 37% was applied in all the prepared surfaces for 30 seconds (Ultraetch, Ultradent Products, Inc.), and the acid was rinsed off with water for 30 seconds. A thin layer of adhesive was applied in the tooth surface (Bottle 2, Adhesive, OptiBond FL, Kerr) (Figure 16), gently air-dried, and light-cured for 20 seconds.

**Figure 17:**
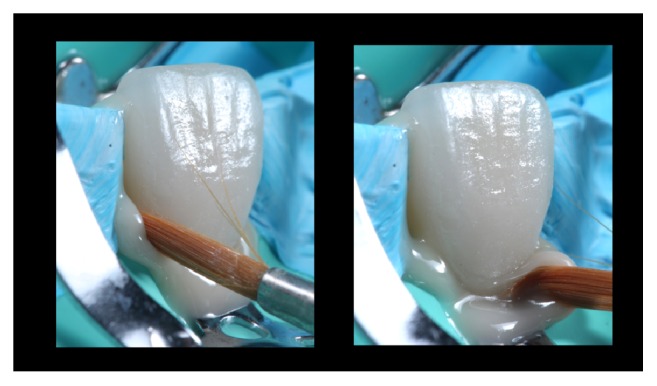
Excess cement removal with a brush. The cement used was value +2 from Variolink Veneer system (Ivoclar Vivadent, Liechtenstein, Germany).

**Figure 18:**
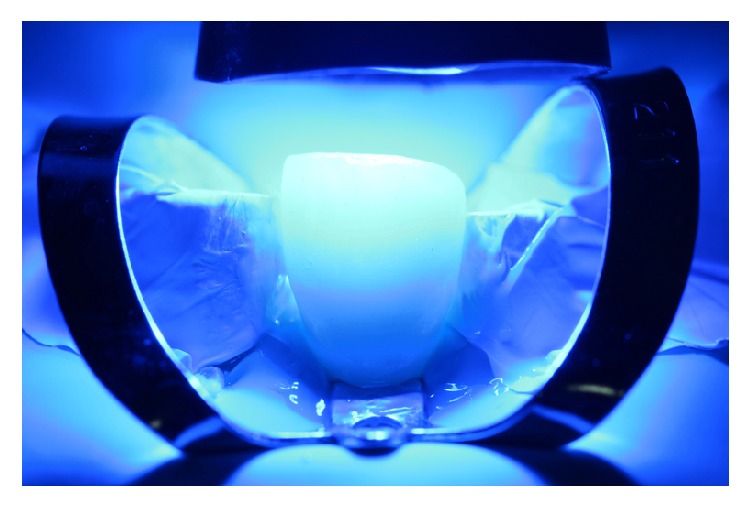
Light curing of the ceramic in position.

**Figure 19:**
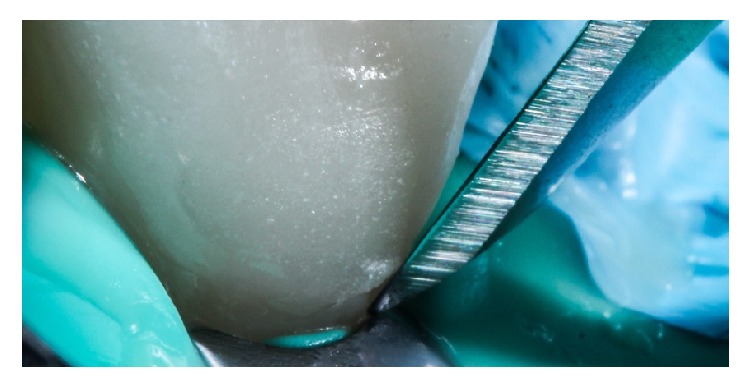
Magnified image showing the elimination of excesses with a number 11 scalpel.

**Figure 20:**
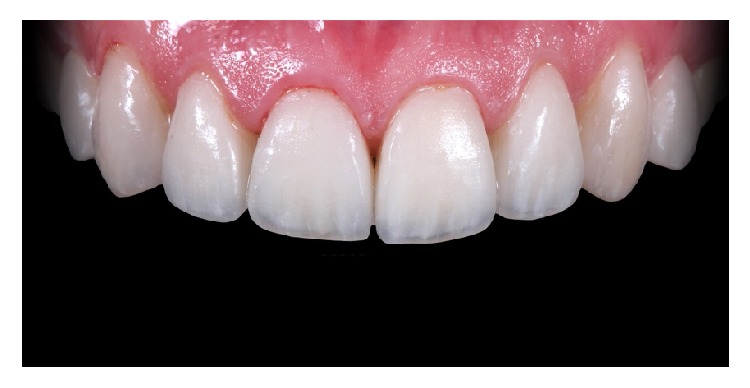
Immediate control image after the cementation of the veneers.

**Figure 21:**
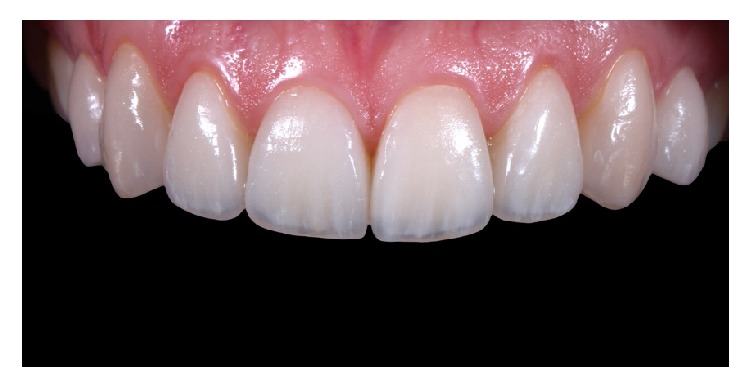
11-month control imagen after treatment. Final result.

The resin cement of choice in this case is a light curing agent, which has no amines. This allows the final color of the cemented restorations to be stable in time. The veneers were taken out from the pad and charged with resin cement in the intaglio surface (Value +2, Variolink Veneer, Ivoclar Vivadent, Liechtenstein, Germany) and then positioned with continuous digital pressure into their correspondent tooth. The excess of cement was removed with a brush (Figure 17) and light-cured for 10 seconds (Bluephase, Ivoclar Vivadent, Liechtenstein, Germany), and this ensures the positioning and fitting of the restoration. Finally, a thin layer of glycerin was putted into the interphase and then light-cured again for 60 seconds in every side of the tooth to ensure the elimination of the oxygen inhibition layer (Figure 18). The final excesses were removed using a scalpel blade number 11 (Figure 19) and then the surface was polished. The immediate result of this clinical case after the cementation is shown in Figure 20. 11-month control pictures are shown in Figure 21.

## 3. Discussion

The present article shows a conservative approach for the management of enamel loss in a predictable and conservative way.

CAD/CAM technology has been a recently incorporated technology in dentistry, reducing the time of fabrication of ceramic restorations; this clinical advantage has allowed clinicians to make chairside restorations in a single session [11, 12]. In this case report, the restoration of the tooth initiates from the digitalization of the wax-up. This information is then tested through the mock-up phase until the final ceramic restorations are fabricated, always keeping the identical shape from the deciding design in every step during the treatment.

Lithium disilicate ceramic veneers processed through CAD/CAM systems are monolithic restorations [3, 13], which means that the final shape of the restoration will be obtained in a single ceramic material and which usually lacks the most common effects of the incisal edge of the anterior dentition such as opalescence, counter-opalescence, different kind of characterizations like white spots, and well-defined mamelon [1].

The advantages of the IPS e.max CAD system includes a reduced number of clinical appointments, with these ceramic veneers being made in one single session (chairside). The variety of translucency blocks (HT: high translucency, LT: low translucency, and MO: medium opacity), colors (20 colors for HT and LT and 5 for MO) and two shapes of blocks according to the manufacturer information allows an extended clinical indication with several restorative possibilities for the anterior region. The flexural strength of the final laminate veneer restoration is approximately 360 MPa [14], which is sufficient to cover the optical and functional needs for this clinical case [15]. Other ceramic CAD-CAM systems based on zirconia-reinforced lithium silicate have been used for the elaboration of laminate veneers [6]. VITA Suprinity® (VITA) and Celtra® Duo (Dentsply) are both two types of glass ceramic with improved mechanical properties, which can also be considered to restore the posterior teeth, but these new ceramics have only two types of translucency (HT and T for VITA Suprinity; HT and LT for Celtra Duo), with less number of colors and shapes of the blocks, having less clinical potential in comparison with the IPS e.max CAD system, even in those cases where the dental substrate must be masked by a medium opacity ceramic.

The selected technique for the veneers finalization corresponds to a layering technique, which allows obtaining a tridimensional effect that simplifies the correction of texture and shape. On the other hand, the staining technique is very common and its real advantage for the characterization and application of stains in the ceramic surface is the ease and speed with which it can be realized; even so, it is a superficial pigmentation, which can be removed if the shape or texture must be corrected in some cases, constituting a great disadvantage. In the layering technique, as the characterization is given by ceramic masses, the modification of the shape or texture of the restoration is not as critical as the staining technique, being the technique of choice for the similarity of natural teeth in this case [16, 17].

Adhesive resin materials, such as those used for cementing laminate veneers, require a dry environment because saliva, blood, and gingival fluids can destabilize the adhesive phase between resin-based materials and tooth structure, either enamel or dentin [18, 19]. In order to prevent this problem, the authors suggest performing rubber dam isolation to avoid this contamination of the enamel bonding surface, allowing a clean restorative environment, with a correct visualization of the gingival margin during the adjustment of the veneer restorations, and finally to facilitate the removal of excess cement as shown in Figure 19.

## 4. Conclusions

Within the limitation of this article, the authors recommend the need to finish monolithic CAD/CAM restorations with nanofluorapatite ceramics to ensure the final esthetic results and the optimal reproduction of details of the characteristics of the incisal third in the anterior teeth.
